# Individual fluid plans versus ad libitum on hydration status in minor professional ice hockey players

**DOI:** 10.1186/s12970-017-0183-x

**Published:** 2017-08-01

**Authors:** Dawn M. Emerson, Toni M. Torres-McGehee, Charles C. Emerson, Teri L. LaSalle

**Affiliations:** 1 University of South Carolina, Department of Physical Education and Athletic Training, 1300 Wheat Street, Blatt PE Center, Room 218, Columbia, SC 29208 USA; 2 Current address: Department of Health, Sport, and Exercise Sciences, University of Kansas, Robinson, Room 161, Lawrence, Kansas, 66045 USA; 3 Current address: Department of Exercise Science, University of South Carolina, 921 Assembly Street, Public Health Research Center, Columbia, SC 29208 USA; 4 Current address: Department of Athletics, University of Missouri-Kansas City, 5100 Rockhill Road, SRC 201, Kansas City, MO 64110 USA; 5 Current address: Hughston Society, Columbus, GA USA

**Keywords:** Hypohydration, Urine specific gravity, Urine color, Body mass loss, Fluid volume

## Abstract

**Background:**

Despite exercising in cool environments, ice hockey players exhibit several dehydration risk factors. Individualized fluid plans (IFPs) are designed to mitigate dehydration by matching an individual’s sweat loss in order to optimize physiological systems and performance.

**Methods:**

A randomized control trial was used to examine IFP versus ad libitum fluid ingestion on hydration in 11 male minor professional ice hockey players (mean age = 24.4 ± 2.6 years, height = 183.0 ± 4.6 cm, weight = 92.9 ± 7.8 kg). Following baseline measures over 2 practices, participants were randomly assigned to either control (CON) or intervention (INT) for 10 additional practices. CON participants were provided water and/or carbohydrate electrolyte beverage to drink ad libitum. INT participants were instructed to consume water and an electrolyte-enhanced carbohydrate electrolyte beverage to match sweat and sodium losses. Urine specific gravity, urine color, and percent body mass change characterized hydration status. Total fluid consumed during practice was assessed.

**Results:**

INT consumed significantly more fluid than CON (1180.8 ± 579.0 ml vs. 788.6 ± 399.7 ml, *p* = 0.002). However, CON participants replaced only 25.4 ± 12.9% of their fluid needs and INT 35.8 ± 17.5%. Mean percent body mass loss was not significantly different between groups and overall indicated minimal dehydration (<1.2% loss). Pre-practice urine specific gravity indicated CON and INT began hypohydrated (mean = 1.024 ± 0.007 and 1.024 ± 0.006, respectively) and experienced dehydration during practice (post = 1.026 ± 0.006 and 1.027 ± 0.005, respectively, *p* < 0.001). Urine color increased pre- to post-practice for CON (5 ± 2 to 6 ± 1, *p* < 0.001) and INT (5 ± 1 to 6 ± 1, *p <* 0.001).

**C**onclusions**:**

Participants consistently reported to practice hypohydrated. Ad libitum fluid intake was not significantly different than IFP on hydration status. Based on urine measures, both methods were unsuccessful in preventing dehydration during practice, suggesting practice-only hydration is inadequate to maintain euhydration in this population when beginning hypohydrated.

## Background

Ice hockey is a fast-paced, equipment-intensive sport played in a cold environment. Athletes perform high intensity, short duration bouts of quick accelerations and decelerations with changes in direction during on-ice shifts lasting 45–90 s [1]. Players rely on both anaerobic force production and aerobic efficiency [2]. It is estimated that players’ on-ice oxygen consumption reaches 80% of their $\dot{\mathrm{V}}$O_2max_ [3]. This type of high intensity exercise increases plasma lactate concentrations, which correlates with increased plasma osmolality and plasma sodium concentration [4] and is known to increase vasopressin [5]. In response to these physiological changes, individuals will voluntarily take in more water after intense exercise [6]. Voluntarily consuming more fluid during exercise can be complicated by factors such as fluid availability, rest time, and other sport dynamics [7]. Further, during intense exercise gastric emptying slows down, causing fluid that is consumed to remain in the stomach [8]. An individual may then limit voluntary intake due to gastrointestinal discomfort. Inadequate fluid consumption, leading to hypohydration, will limit the amount of fluid available to maintain physiological function (e.g. heart rate and blood pressure) during exercise.

The equipment and cold environment in ice hockey also influences hydration in athletes. The protective equipment required in ice hockey increases core body temperature, sweat loss, and plasma lactate; thus, resulting in decreased power output and compromising fluid balance [9, 10]. Athletes in cool environments are often unable to replace enough fluid to counter sweat losses [9, 11–13]. In part, this is due to a blunted thirst response seen with moderate-intense (70% $\dot{\mathrm{V}}$O_2max_) exercise [14]. Individuals reduce the amount of fluid they consume, resulting in voluntary dehydration. Additional confounding factors for fluid intake in the cold include cold-induced diuresis and an increase in insensible sweat and respiratory fluid loss [15, 16].

In contrast to voluntary dehydration, some individuals consume more fluid than necessary and gain weight by the end of exercise, even in the cold [14]. To discourage excessive or inadequate fluid consumption, the American College of Sports Medicine and National Athletic Trainers’ Association recommend individualized fluid plans (IFPs) [7, 17]. IFPs use an individual’s distinct sweat rate to guide adequate fluid replacement during exercise and maintain euhydration. There is some speculation to how 2% body mass (BM) loss may impact an individual’s health and performance, with some evidence that there is no detriment to performance [8, 18] and others showing aerobic and anaerobic impairment [19, 20]. Regardless, it is generally recommended to maintain less than 2% loss from pre- to post-exercise [7, 17]. In clinical settings, 2% loss may not represent 2% from a euhydrated baseline BM. For instance, an athlete beginning practice hypohydrated who loses 2% during practice is at a larger deficit by post-practice. This is an important consideration because many ice hockey players begin activity hypohydrated [11–13, 21, 22].

The environmental conditions, complex physical demands, and protective equipment associated with ice hockey heightens the importance of proper hydration to maintain health and performance. Most research examining hydration in ice hockey is limited to 1–4 days [11, 12, 22] and utilizes junior and collegiate players [9, 11–13]. Often, fluid intake did not match sweat rate, resulting in dehydration [9, 11, 12, 22]. Existing literature on the effects of matching fluid consumption to sweat rate is primarily in controlled laboratory settings [8, 18]. Field studies investigating the effectiveness of IFPs versus ad libitum are limited [21, 23] and have not included sweat sodium concentration ([Na^+^]). The purpose of this study was two-fold: 1) to implement and determine efficacy of IFPs vs ad libitum fluid consumption in the field setting of an ice hockey team and 2) to examine fluid and electrolyte balance in minor professional ice hockey players across multiple practices. We hypothesized that providing IFPs would improve pre- and post-practice hydration measures compared to ad libitum. We also hypothesized that players in the ad libitum group would report to practices hypohydrated and remain hypohydrated at post-exercise.

## Methods

We utilized a randomized controlled design to compare 2 experimental conditions: control (CON) and intervention (INT) on hydration status. Our main outcome measures were urine specific gravity (Usg), urine color (Ucol), urine sodium concentration ([Na^+^]), urine potassium concentration ([K^+^]), and percent body mass change (%BM). We also assessed fluid volume (Fvol) and sodium ingestion. Data collection occurred at 10 practices over 3 months during the middle of the hockey season (November – January) to avoid pre-season physiological changes and allow rosters to be set. To minimize game effects and differences in practice intensity, no data collection was scheduled on days immediately before or after a game.

### Participants

Twenty-one male minor professional ice hockey players consented to participate. We experienced attrition due to trades, call-ups, and voluntary cessation, yielding 11 participants. Three participants had incomplete data; therefore, we conducted analysis on a total of 8 participants (2 forwards, 4 defensemen, and 2 goalies; mean age = 24.4 ± 2.6 years, height = 183.0 ± 4.6 cm, weight = 92.9 ± 7.8 kg, and body fat = 14.0 ± 3.8%). Participants were required to be in good general health. Participants were free of cardiovascular, respiratory, and metabolic disorders; fluid and electrolyte balance disorders; and gastrointestinal and swallowing disorders. Prior to participation, participants signed an informed consent form approved by the Institutional Review Board at the University of South Carolina (#HSA4821). Participants were asked to maintain normal levels of physical activity, maintain a normal state of hydration, and avoid changes in caffeine consumption and dehydration-inducing substances (e.g., alcohol) during the course of this study.

Baseline measures were assessed over 2 practices. Participants’ pre- and post-practice BM, Usg, Ucol, and urine [Na^+^] and [K^+^] were measured. Practice sweat [Na^+^] and sweat rate were also measured. At the end of baseline, participants were randomly assigned to one of the 2 conditions (CON or INT). Total Fvol consumed was measured during practice by providing individual water bottles identifiable by the player’s assigned team number. To minimize crossover between participants and non-participants, participants’ bottles were a different color and located separately from non-participants’ bottles.

The CON condition required participants maintain normal hydration habits (i.e., drinking ad libitum) during practice. Participants received either one water (W), one commercially available carbohydrate-electrolyte beverage (CEB; Powerade, The Coca-Cola Company, Atlanta, GA) or one of each, depending on individual preference. CON participants were not aware of their individual needs (based on sweat rate and [Na^+^]) or the INT participants’ fluid needs.

Each INT participant was provided individualized recommendations regarding Fvol to consume per hour of practice that corresponded to his baseline sweat rate and sweat [Na^+^]. The INT group received one bottle with W and one electrolyte-enhanced CEB (E-CEB). For example, if a participant’s baseline sweat rate = 2 L/h and his sweat [Na^+^] = 1000 mg/L/h, an E-CEB was made to match the sodium loss. The E-CEB contained a measured amount of commercially available electrolyte mixture (Gatorlytes, Gatorade Company, Inc., Chicago, IL; sodium = 780 mg/packet) combined with the CEB (sodium = 150 mg/L). In the example, the participant would be given 1 L of CEB mixed with 1 Gatorlyte packet for a total of 930 mg of sodium. He would also be provided 1 L of water. Prior to practice starting, the player was verbally recommended to consume both bottles of fluid during practice and within 1 h (average practice length). To minimize interference during practice, no verbal instructions were given to players during practice. Participants were allowed to consume more than recommended. All bottles were continuously checked throughout practice and refilled, as necessary, with a pre-measured fluid amount. Fluid remaining in bottles at the end of practice was measured using a graduated cylinder and recorded. Samples were taken from each CEB to determine beverage [Na^+^]. Sodium concentration was corrected for participant’s ingested Fvol to determine total sodium ingested.

### Instruments and protocols

#### Hydration indices

Hydration status was characterized using a Ucol chart (Human Kinetics, Champaign, IL), Usg (refractometer model REF 312, Atago Company Ltd., Tokyo, Japan), and %BM (Tanita TBF-300A, Tanita Corporation, Tokyo, Japan). Dehydration was defined as >1% BM loss from pre-practice weight, a Usg ≥ 1.020, and/or Ucol shade ≥4 [7].

#### Electrolytes

Sweat and urine electrolytes were assessed using ion-selective electrode analysis (EasyLyte® Na^+^/K^+^ electrolyte analyzer, Medica, Bedford, MA). Normal urine [Na^+^] was defined as 40–220 mmol/L and normal urine [K^+^] as 25–120 mmol/L. Participants were considered to be in a state of sodium conservation when urine [Na^+^] < 40 mmol/L.

##### Sweat

Prior to the participant dressing for practice, a sweat patch was applied to the forearm. Researchers wore rubber gloves, cleaned the forearm area with alcohol and placed a 3 × 3 sterile gauze pad on the area. The gauze pad was held in place with a water resistant clear adhesive patch (SEAL-TIGHT® Shower Patch™). Immediately after practice, researchers removed patches and sweat was squeezed into a 2 ml microtube, closed, placed on ice, and set aside to be analyzed.

##### Environment

Temperature and relative humidity were monitored with an environmental monitor (QuestTemp-34, QuestTechnology, Oconomowoc, WI) to determine practice arena environment.

#### Body composition

On two occasions, once at baseline (November) and once at the end of the data collection (January), body fat % was calculated using a 7-site (i.e., chest, mid-axillary, triceps, subscapular, abdomen, suprailiac, and thigh) skin fold procedure with Lange skinfold calipers (Beta Tehnology Inc., Cambridge, MD).

### Statistical analysis

We used IBM SPSS 22 (IBM, Inc., Chicago, IL) for all analyses with a significance level of *p* < 0.05. Descriptive statistics were calculated for all dependent measures. A 2 (condition) × 9 (practice days) repeated measures ANOVA was utilized to determine changes in dependent measures. Day 2 was excluded due to a team function preventing post-practice data collection. One-way ANOVAs and paired sample t-tests were used to determine differences in demographics, baseline measures, hydration and electrolytes, Fvol, and sodium consumed between and within CON and INT. Due to missing data, only 7 practice days were used to analyze urine [Na^+^] and [K^+^]. Post-hoc power analysis using recommended versus actual Fvol and pre- to post-Usg indicated a statistical power > 0.70.

## Results

There were no significant differences in age, baseline height, weight, or body fat % between groups (Table 1). Body mass and body fat % did not significantly change from baseline to the end of the data collection period (3 months). Mean practice time for 10 days = 68.4 ± 14.8 min (range = 50–78 min). Mean arena temperature = 12.6 °C and relative humidity = 47%. Baseline mean sweat rate (3.4 ± 1.4 L/h) and sweat [Na^+^] (63.1 ± 17.1 mmol/L; 145.0 ± 39.3 mg/L) were not significantly different between conditions. Sweat rate and [Na^+^] did not significantly differ during practices between CON (2.1 ± 0.5 L/h and 54.3 ± 22.3 mmol/L) and INT (2.5 ± 0.9 L/h and 70.2 ± 19.5 mmol/L).

**Table 1:** Participant demographics (M ± SD)

	Control (*N* = 4)	Intervention (*N* = 4)
Age (yr)	25.5 ± 2.6	23.3 ± 2.2
Height (cm)	182.8 ± 5.4	183.3 ± 4.4
Weight (kg)	91.8 ± 8.0	94.0 ± 8.7
Body fat (%)	13.1 ± 3.6	14.9 ± 4.2

No significant differences between conditions

On average, the INT maintained <1% BM loss during practice and there was no significant difference from CON (Table 2). Mean Usg and Ucol indicated participants in both groups were hypohydrated before practice and experienced significant dehydration during practice (Table 2). There were no significant differences between CON and INT Usg at pre- or post-practices for any days.

**Table 2:** Pre- and post-practice hydration and electrolyte measures by condition (M ± SD)

	Control		Intervention	
	Pre	Post	Pre	Post
Urine specific gravity	1.024 ± 0.007	1.027 ± 0.005^*a*^	1.024 ± 0.006	1.026 ± 0.006^b^
Urine color	5 ± 2	6 ± 1^*c*^	5 ± 1	6 ± 1^*d*^
% Body mass loss	-	−1.14 ± 0.68	-	−0.95 ± 0.68
Urine sodium (mmol/L)	88.3 ± 56.4	72.3 ± 44.7	81.5 ± 48.8	73.5 ± 44.4
Urine potassium (mmol/L)	45.5 ± 23.9	80.6 ± 40.2^*e*^	65.1 ± 38.1^f^	83.1 ± 42.6^*g*^

Euhydration and normal electrolyte values: urine specific gravity = < 1.020, urine color <4, body mass loss <1%, urine sodium 40–220 mmol/L, and urine potassium 25–120 mmol/L

^a^Significantly greater than pre-practice (*t*(35) = −2.8, *p* = 0.008, *d* = −0.5)

^b^Significantly greater than pre-practice (*t*(35) = −2.3, *p* = 0.031 *d* = −0.4)

^c^Significantly greater than pre-practice (*t*(35) = −4.9, *p* < 0.001, *d* = −0.8)

^d^Significantly greater than pre-practice (*t*(35) = −3.9, *p* < 0.001, *d* = −0.7)

^e^Significantly greater than pre-practice (*t*(25) = −5.1, *p* < 0.001, *d = −*1.0)

^f^Significantly greater than Control pre-practice (*F*_1,50_ = 5.0, *p* = 0.030, ω^2^ = 0.1)

^g^Significantly greater than pre-practice (*t*(23) = −2.6, *p* = 0.016, *d = −*0.5)

Overall mean total fluid, CEB, and sodium intake was significantly higher in the INT (Table 3). Both CON and INT consumed significantly less fluid and sodium than recommended based on sweat rate and [Na^+^] (Table 3). CON participants replaced 25.4 ± 12.9% of their fluid needs and INT 35.8 ± 17.5%. Looking at individual data, no participant consumed fluid or sodium on any practice day matching recommended intake. When corrected for BM, fluid was consumed at a rate of 7.5 ml/kg/h for CON and 11.0 ml/kg/h for INT.

**Table 3:** Recommended versus actual fluid and sodium consumed (M ± SD)

Condition	Recommended Intake^a^		Actual Consumed during Practice				
	Fluid (L/h)	Sodium (mg/L)	Water (L)	CEB (L)	Total Fluid (L)	Sodium (mg)	Fluid Rate (L/h)
Control	3.1 ± 0.7	115.8 ± 42.8	0.7 ± 0.5	0.06 ± 0.1	0.8 ± 0.4^b^	4.2 ± 8.4^c^	0.7
Intervention	3.3 ± 0.8	162.1 ± 30.1	0.5 ± 0.4^d^	0.7 ± 0.5^e^	1.2 ± 0.6^f,g^	95.0 ± 46.6^h,i^	1.0

*Abbreviation*: *CEB* carbohydrate-electrolyte beverage

^a^Based on baseline sweat rate and sweat sodium concentration

^b^Significantly less than recommended intake (*t*(3) = 5.6, *p* = 0.011, *d* = 2.8)

^c^Significantly less than recommended intake (*t*(3) = 4.4, *p* = 0.022, *d* = 2.2)

^d^Significantly less than Control (*F*_1,63_ = 6.7, *p* = 0.012, ω^2^ = 0.1)

^e^Significantly greater than Control (*F*_1,66_ = 67.5, *p <* 0.001, ω^2^ = 0.5)

^f^Significantly greater than Control (*F*_1,66_ = 10.7, *p* = 0.002, ω^2^ = 0.1)

^g^Significantly less than recommended intake (*t*(3) = 5.4, *p* = 0.013, *d* = 2.7)

^h^Significantly greater than Control (*F*_1,6_ = 14.8, *p* = 0.009, ω^2^ = 0.6)

^i^Significantly less than recommended intake (*t*(3) = 7.8, *p* = 0.004, *d* = 3.9)

Urine [Na^+^] did not significantly change from pre- to post-practice for CON or INT and there was no significant differences between conditions (Table 2). In the INT, participants reported 23% of the time with a urine [Na^+^] < 40 mmol/L (mean = 23.2 ± 2.5 mmol/L) and 24% ended practice in a similar state (18.9 ± 14.6 mmol/L). Similar results were found for the CON, with 26% reporting with a low urine [Na^+^] at pre-practice (29.5 ± 7.6 mmol/L) and 31% at post-practice (27.4 ± 9.0 mmol/L). Post-practice urine [K^+^] was significantly greater than pre- for both conditions (Table 2). Only once (4% of the time) did an INT participant arrive to practice with urine [K^+^] < 25 mmol/L (22.2 mmol/L), compared to18.5% of the time in the CON (17.1 ± 5.6 mmol/L). No participant had low urine [K^+^] post practice. High urine [K^+^] (>120 mmol/L) occurred 16% of the time within CON (156.2 ± 19.1 mmol/L) and 12.5% in INT (164.0 ± 44.7 mmol/L).

Three of four CON participants regularly consumed only W during practice. These 3 participants had significantly higher sweat [Na^+^] than the fourth CON participant (136.4 ± 13.7 versus 53.7 mmol/L, *t*(2) = 17.3, *p* = 0.003, *d* = −6.0) and more frequently their urine [Na^+^] was <40 mmol/L (31% versus 14%). Despite consuming an E-CEB, the 2 INT participants with higher sweat [Na^+^] most frequently had urine [Na^+^] < 40 mmol/L (33%) compared to the other 2 INT participants (0.8%).

## Discussion

We assessed fluid and sodium balance in minor professional ice hockey players and examined these variables after implementing IFPs. We found players reported to practice hypohydrated and experienced negative fluid and sodium balance during practice. Once players in the INT group were informed of their IFP, participants consumed more fluids and electrolytes than the players drinking ad libitum. Despite increased fluid ingestion, there were no significant differences between conditions for hydration and sodium measures. Based on urinary hydration measures, the IFP was unsuccessful in offsetting fluid loss during ice hockey practice. Further, IFP participants were unable to consume the recommended Fvol.

### Fluid and sodium balance

Despite playing in cool environments, ice hockey players are susceptible to dehydration [9, 11–13, 22], increased core temperature [9], and sodium depletion [11–13]. Our mean sweat rates were higher than collegiate (0.8 L/h) [9] and elite junior ice hockey players (1.5 ± 0.1 L/h and 1.8 ± 0.1 L/h) [12, 13] yet similar to professional athletes in warmer environments [24, 25]. Similarly, our participants’ mean sweat [Na^+^] was comparable to other ice hockey studies and professional football [11–13, 24]. Despite the higher sweat rates, we observed BM losses similar to collegiate practices (−1.1%) [9], juniors practice (−0.8%) [12], and a juniors game (−1.3%) [11]. In order to maximize performance and maintain health, it is advisable to develop a hydration strategy to limit fluid losses to less than 2%.

We did not expect to find our IFP participants repeatedly arriving to practice hypohydrated. However, our results are consistent with previous ice hockey studies [11, 12, 22] as well as other sports [24]. Pre-exercise Usg has not correlated with ad libitum fluid consumption during activity [11], having very little effect on hydration-related hormonal and vascular responses during exercise when fluid is available [26]. In other words, athletes observed in training and competition do not tend to consume more fluids when beginning exercise hypohydrated. It is important to note using %BM calculations as the only hydration measure may not accurately portray an individual’s fluid balance and can underestimate actual hypohydration level. This concept is reflected in our study, and supported by Cleary et al. [21], where participants experienced <2%BM loss with an IFP, but frequently began practice hypohydrated and experienced significant dehydration during activity. To prevent dehydrating during activity and chronic hypohydration, athletes need individualized hydration monitoring and fluid replacement recommendations that extend beyond practice only.

Urine electrolyte excretion can vary depending on diet. Although we were unable to determine dietary influences, the frequency of low urine [Na^+^] among those only consuming water and those with higher sweat [Na^+^] suggests these individuals were often in a state of sodium conservation. This is supported by the increase in urine [K^+^] from pre- to post-practice. There are few field studies examining urine electrolytes, but our results are consistent with professional American football players completing 2-a-day practices in hot environments [27]. It is possible the elevated post-practice potassium was a result of potassium leaking from working and/or damaged muscle during intense exercise. However, to maintain electrolyte balance, increased urine potassium often accompanies decreased urine sodium as the kidneys use sodium to retain water during hypohydration [28]. In addition to matching sweat rates, individuals with high sodium losses or low sodium intake (e.g., low-sodium diet) receive more specific sodium recommendations that can come with an IFP.

### Individualized fluid plans

Voluntary dehydration commonly occurs in athletes [9, 11–13] despite unlimited access to water. Individuals who plan to purposefully consume more fluid during exercise increase ad libitum consumption compared to those without purposeful intentions [29]. Using a similar theoretical approach, we informed INT participants on individual baseline data and gave target Fvol to meet during practice. This is in contrast to adolescent volleyball players provided metered IFPs to match fluid loss [21] and runners provided designated water stations with specific fluid requirements throughout a 20-km race [23]. Though fluid consumption increased, to our surprise, IFP participants continued to experience dehydration, suggesting inadequate fluid absorption during practice. The two most significant factors affecting fluid absorption rate and availability are gastric emptying and intestinal absorption [30]. Some INT participants were provided E-CEBs to match higher sweat [Na^+^], which increased beverage [Na^+^] and osmolarity. Research has shown gastric emptying is not affected by CEB [Na^+^] or osmolarity [31]. Therefore, it is unlikely beverage composition attenuated fluid absorption and negatively affected post-practice hydration measures.

Two factors that may have hindered fluid absorption are practice length and intensity. Mean practice length in the present study was similar to Palmer et al. [12], but shorter than other hockey studies [9, 13]. During a 60 min cycling time-trial in which participants replaced 100% of sweat losses, of the ingested 1.5 L, 0.3 L remained in the stomach and 0.2 L was urinated out following exercise. Only 1.02 L was available for fluid replacement [8]. If gastric emptying was slowed due to the high practice intensity, our participants may have voluntarily limited fluid consumption in fear of causing gastrointestinal discomfort. Ingesting fluid volumes, even water, matching 100% sweat loss may induce feelings of fullness and bloating [18]. We believe the short practice length and high intensity made it difficult for participants to absorb and utilize some of the ingested fluid, and may partially explain the similar post-practice hydration measures between groups.

#### Limitations and future research

A unique aspect of our study is the cross-sectional look at mid-season hydration rather than a one day snap shot or several days over one week. Unfortunately, because of this approach we experienced high participant attrition over the 3 months. Though similar to previous research [10], our sample size is a limitation. The higher mean baseline sweat rate compared to mean practice sweat rates is likely due to the conditioning activity at the end of practices earlier in the season compared to practices later in the season. Using a one-site local sweat patch is a limitation because of the tendency to overestimate sweat [Na^+^]. Whole-body wash down is a more valid technique to assess sweat [Na^+^]. Using urine [Na^+^] and [K^+^] only allows for generalizations on electrolyte balance. Measuring plasma electrolytes and fluid regulatory hormones (e.g., aldosterone) is optimal, but collecting blood samples was impractical in this field setting. Measuring plasma volume or osmolality would provide a more accurate indication of hydration status. However, one time urine collection (i.e., pre-practice) to determine hydration status, although less valid, is a common, practical tool for clinicians. We did not encourage participants to void urine upon waking and prior to coming to practice. Morning Usg measures are often skewed high. Obtaining a subsequent urine sample would provide a more accurate Usg measure. Practices occurred mid-morning and we assume some participants voided before coming to practice. The purpose of this study was to examine an IFP during practice; therefore, we did not provide participants with recommendations for post-practice hydration to attenuate losses. Only examining hydration and fluid during practice limited our ability to identify daily nutritional factors that may positively or negatively affect hydration in this population. Future research should consider dietary analysis and 24 h urine collection to better describe these individual’s hydration state. We did not formally measure gastrointestinal discomfort. No participant reported emesis or gastrointestinal distress during the IFP. However, a number of participants verbally reported during baseline testing that they generally did not consume CEB during practice due to gastrointestinal symptoms (i.e., fullness and bloating). Future studies are warranted to examine gastrointestinal distress with IFPs. Finally, we did not control for individual exercise intensity during practice, which may have affected Fvol.

Interestingly, one participant in the INT group, a goalie, reported a history of exercise associated muscle cramps, dehydration, and fatigue during long games, particularly in arenas in the Southeast. Individual results from this participant showed he had the highest sweat rate, sweat [Na^+^], fluid ingestion and frequently lower urine [Na^+^] compared to any other participant. Research should continue to examine risk factors (e.g., equipment, environment, travel, etc.) and symptoms of fluid and thermoregulatory imbalance in ice hockey players.

## Conclusions

Our primary purpose was to determine if an IFP would improve hydration status compared to ad libitum fluid intake among male, minor professional ice hockey players. Despite increased fluid intake and attenuated BM loss, IFPs did not improve hydration status during mid-season ice hockey practices. When developing IFPs, clinicians should consider individuals completing a short, high intense activity may be unable to consume enough fluid to match losses. Since participants reported to practice hypohydrated, it is likely daily hydration behaviors were inadequate to maintain fluid balance and not influenced by the IFP. Members of the sports medicine team should monitor hydration status and educate ice hockey players on proper pre- and post-activity hydration to maintain daily fluid balance and limit negative dehydration induced performance and health effects during activity.

## Abbreviations

BM: Body mass
CEB: Carbohydrate electrolyte beverage
CON: Control
E-CEB: Electrolyte enhanced carbohydrate electrolyte beverage
Fvol: Fluid volume
IFP: Individual fluid plans
INT: Intervention
Ucol: urine color
Usg: urine specific gravity
$\dot{\mathrm{V}}$O_2max_: maximum volume of oxygen consumed
W: water
